# Hierarchical Structure of Cellulose Nanofibril-Based Foams Explored by Multimodal X-ray Scattering

**DOI:** 10.1021/acs.biomac.1c00521

**Published:** 2022-02-23

**Authors:** Viviane Lutz-Bueno, Ana Diaz, Tingting Wu, Gustav Nyström, Thomas Geiger, Carlo Antonini

**Affiliations:** † Paul Scherrer Institute, 5232 Villigen, Switzerland; ‡ Department of Health Sciences and Technology, ETH Zürich, 8092 Zürich, Switzerland; § Laboratory for Cellulose and Wood Materials, Empa Swiss Federal Laboratories for Materials Science and Technology, 8600 Dübendorf, Switzerland; ∥ Department of Materials Science, University of Milano-Bicocca, 20126 Milano, Italy

## Abstract

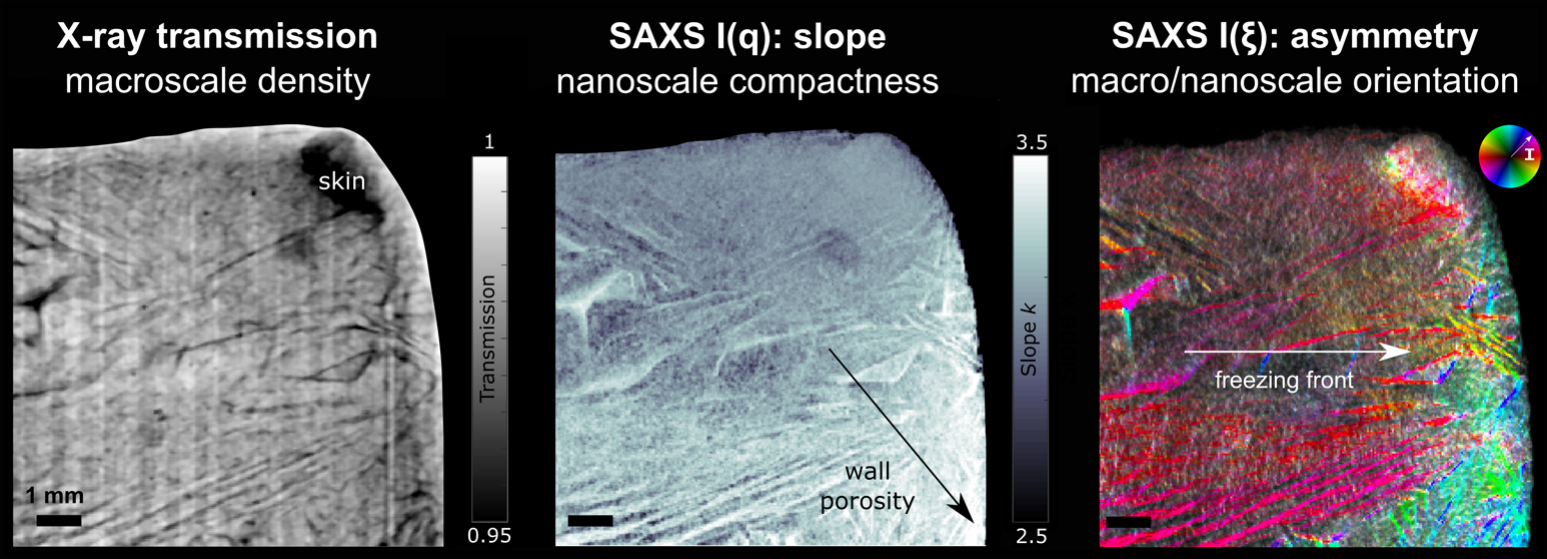

Structural characterization techniques are fundamental to correlate the material macro-, nano-, and molecular-scale structures to their macroscopic properties and to engineer hierarchical materials. Here, we combine X-ray transmission with scanning small- and wide-angle X-ray scattering (sSWAXS) to investigate ultraporous and lightweight biopolymer-based foams using cellulose nanofibrils (CNFs) as building blocks. The power of multimodal sSWAXS for multiscale structural characterization of self-assembled CNFs is demonstrated by spatially resolved maps at the macroscale (foam density and porosity), at the nanoscale (foam structural compactness, CNF orientation in the foam walls, and CNF packing state), and at the molecular scale (cellulose crystallite dimensions). Specifically, we compare the impact of freeze–thawing–drying (FTD) fabrication steps, such as static/stirred freezing and thawing in ethanol/water, on foam structural hierarchy spanning from the molecular to the millimeter scale. As such, we demonstrate the potential of X-ray scattering imaging for hierarchical characterization of biopolymers.

## Introduction

Cellulose is the most abundant biopolymer on the planet,^1^ and its use by mankind dates back to ancient civilizations.^2^ Nonetheless, the use of cellulose has been experiencing a new renaissance in the past decade.^3^ On one hand, cellulose-based materials represent a sustainable alternative to fossil-based polymers, because of their high availability, biodegradability, and low costs.^4^ On the other hand, novel material manipulation and characterization techniques at the nanoscale have enabled the development of cellulose-based nanomaterials.^5−7^

Cellulose is a polysaccharide composed of β(1→4)-linked d-glucose monomers linked by hydrogen bonds. The biopolymer chain forms bundles of nanofibrils, in which highly ordered regions, called the crystalline phase, alternate with disordered regions, called the amorphous phase. In particular, cellulose nanofibrils (CNFs), also referred to as nanocellulose, show enhanced mechanical properties at the nanoscale.^8,9^ CNFs can assemble into diverse structures and are prospective building blocks for designing novel hierarchical functional materials with outstanding properties. CNF-based materials span from films, e.g., edible packaging,^10^ food sensing,^11^ antioxidant barrier,^12^ and membranes for reverse osmosis,^13^ to complex 3D printed shapes with tunable orientation and structure.^14,15^ Additionally, CNF is an attractive biomaterial^5,16^ that is used in wound-healing applications,^17^ in antibacterial surfaces,^18,19^ and even as anticoagulants.^20^ Here, we focus on the high strength-to-weight ratio of CNFs, which is ideal for developing high-performance porous lightweight foams,^21^ either pure or in combination with other biopolymers^5^ and/or nanoparticles.^22−24^ Such high-performance foams must have high surface areas and low densities, properties that define their performance for gas adsorption,^25^ thermal insulation,^6,26^ selective liquid absorption for environmental remediation,^18,23,27,28^ and energy harvesting.^24^

In parallel to lab-scale characterization and design of functional CNF-based materials, increasing efforts are devoted to enabling the scalability of the cellulose fibrillation process, as well as to the large-scale production of CNF-based materials. Recently, we reported a facile and scalable freeze–thawing–drying (FTD) process, developed to fabricate CNF-based foams.^29^ FTD is based on ice-templating, a multistep process, in which an aqueous CNF suspension freezes, while ice crystals provide a negative template for CNFs to assemble into the foam skeleton.^30^ When the ice is removed, an open-pore foam structure composed of aggregated CNFs should remain. Vacuum-drying is often used to directly sublimate the ice, forming foam structures with high porosity and low densities^31−35^ in the range of 5–60 mg/cm^3^. Freeze-drying based on vacuum, is one of the most widely used powder drying techniques in the industry, together with spray-drying and fluid-bed techniques, for its high yield.^36^ However, vacuum-drying can be highly energy-demanding and not suitable for large-scale materials.^37^ Instead, FTD includes the thawing step for solvent exchange before drying, which helps to achieve a low foam density (down to 10 mg/cm^3^) and a high foam porosity (up to 99.4%). This thawing step includes solvent exchange in either ethanol or water. The subsequent drying process is particularly critical because capillary forces can lead to structure shrinkage or even to its collapse. Thawing in ethanol reduces capillary forces during drying; however, water is more suitable for large-scale production, for safety and sustainability reasons.

These lightweight and porous CNF-based foams assemble in hierarchical structures that span from the molecular to the millimeter scale. As a consequence, their structural characterization needs to correlate macro-, nano-, and molecular-scale features to the macroscopic mechanical properties and functionalities.^7,38−41^ Scanning X-ray microscopy, such as scanning small- and wide-angle X-ray scattering (sSWAXS), can gather structural information at spatial resolutions ranging from several millimeters to a few angstroms in both real and reciprocal space, covering the gap between microscopy and diffraction.^42,43^ Experimentally, X-ray scatterings in both the small angles (SAXS) and wide angles (WAXS) are similar to X-ray diffraction, which normally provides structural information at the molecular scale. However, SAXS uses much greater sample-to-detector distances, providing information at lower angles and allowing periodicities, particle sizes, and interactions to be investigated at the nanoscale. In synchrotrons, brilliant beam sources and fast acquisition times enable scanning of large sample areas, and the obtention of spatially resolved structural information in the macroscale. Additionally, this brilliance allows beam sizes potentially down to the submicron scale, which determines the spatial resolution of the recorded images; this high resolution is transferred to the reciprocal space and enables nanostructural investigation.

Here we employ sSWAXS to gain understanding of the macro-, nano-, and molecular-scale properties of cellulose nanofibril foams. We focus on how the hierarchical self-assembled structure of CNFs affects the large-scale material behavior, to elucidate how the different fabrication steps of FTD affect the foam structure. From a single multimodal sSWAXS data set, we systematically analyze the morphology of CNF-based foams at the macroscale, such as the foam-wall porosity, the CNF orientation at the nanoscale, and the CNF crystallite dimensions at the molecular scale. Apart from such multiscale structural characterization, sSWAXS enables measurements with high statistics, as large sample areas are scanned while averaging the signal within the sample volume, which is illuminated by the X-ray beam. These material-radiation interactions in a scanning manner provide structural information with high statistics, in a way in which other techniques, such as electron microscopy, often fail. As such, sSWAXS is an ideal technique to simultaneously investigate complex hierarchical structures at different length scales. Because of its potential, we emphasize the power of X-ray scattering imaging in the structural characterization of hierarchical materials for the biopolymer community, using CNF-based foams as a representative case study.

## Materials and Methods

### Production and Characterization of Cellulose Nanofibrils

**Figure 1:**
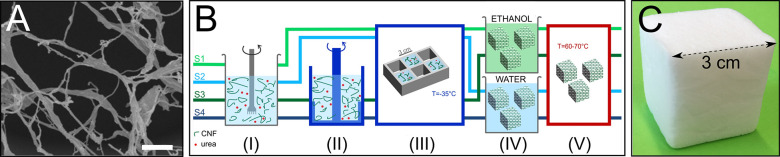
(A) SEM of cellulose nanofibrils. Scale bar: 1 μm. (B) Different foam-fabrication routes by freeze–thawing–drying (FTD) at room pressure. The static freezing process (samples S1 and S2) consists of (I) preparation of an aqueous cellulose nanofibril (CNF) and urea suspension; stirred (II and III) or static (III) freezing; (IV) thawing and washing (in ethanol or water); and (V) oven-drying. Samples S1 and S2 are frozen in static conditions (III), whereas samples S3 and S4 are frozen in two steps (II and III). For S1 and S3, thawing is performed in ethanol, whereas for S2 and S4, thawing is performed in water. Adapted from ref (29). Copyright 2019 MDPI. (C) Final CNF-based foam (cube side ≈ 3 cm). In this study, foams with a porosity in the range of 96–98% were investigated.

Cellulose boards (Schattdecor AG, Germany) were swollen in water and mechanically ground by an ultrafine friction grinder (Supermasscolloider, MKZA10-20J CE, Masuko Sangyo Co., Ltd., Kawaguchi/Saitama, Japan) to obtain an aqueous CNF suspension. The CNF-specific surface area was determined by means of the Brunauer–Emmett–Teller (BET) method.^29^ Nanofibrils were supercritically dried (Quorum Technologies E3100, Laughton, U.K.) and solvent-exchanged from water to liquid CO_2_ via ethanol (10 °C and 50 bar), followed by drying at supercritical conditions (35 °C and 100 bar). Dried nanofibrils were degassed at 105 °C for 4 h before performing nitrogen-sorption measurement (SA3100, Beckman Coulter, Indianapolis, IN, U.S.A.). Figure 1A shows a representative scanning electron microscopy image (SEM, Fei Nova Nanosem 230 Instrument, Fei, Hillsboro, OR, U.S.A.) of the produced CNFs (see ref (29) for details on sample preparation).

### CNF-Based Foam Fabrication

CNF-based foams were produced using a freeze–thawing–drying (FTD) process, described in detail by Antonini et al.^29^ Four variations of the production routes were used to investigate the effects of the freezing mode (static versus stirred freezing) and of the solvent used for thawing (ethanol versus water). The routes are schematically represented in Figure 1B, and the corresponding sample details (from S1 to S4) are summarized in Table 1. Briefly, the production process followed steps I to V. In step I, CNFs and urea, both at a concentration of 2.5 wt %, were suspended in water using a dispersing machine (Ultra-Turrax, IKA). Freezing was performed under either static (step III) or stirred (steps II and III) conditions. In static freezing (samples S1 and S2, step III), the CNF/urea suspension was directly molded to 33 mL cubes and stored at −35 °C for at least 3 h to ensure complete freezing. In stirred freezing (samples S3 and S4, steps II and III), a commercial ice cream machine (Unold, model 48845) was used to create a partially frozen suspension (∼50% of the water, step II) with a more homogeneous ice crystal distribution; the partially frozen suspension was then molded and fully frozen (step III). In step IV, both types of samples were thawed in denatured ethanol (95% ethanol and 5% isopropanol) or in water at room temperature. As seen in Figure 1B, samples S1 and S3 were thawed in ethanol, whereas samples S2 and S4 were thawed in water. In the final step V, foams were dried in a ventilated oven at 65 °C for 3 h to ensure complete solvent evaporation. The use of organic solvents, such as isopropyl alcohol^5^ and ethanol,^29^ significantly reduced the foam capillary collapse upon drying and enabled the fabrication of foams with high porosity (Figure 1C). Foams possessed a characteristic porosity imparted by ice-templating, with a pore size on the order of 100 μm, corresponding to the characteristic size of the formed ice crystals.^29^ As observed previously,^29^ the use of urea affected the freezing process, resulting in a higher final porosity after drying, for a 1:1 CNF–urea ratio. Zawko and Schmidt have already demonstrated that in situ urea dendritic crystal growth can be used to create biopolymer hydrogels with dendritic pore connectivity.^44^ A study of the cellulose–urea–water interaction during nucleation may require specific investigation of the frozen sample structure which goes beyond the scope of this work and is not addressed here.

**Table 1:** Selected Samples

name	solvent	method	wt % CNFs	wt % urea
S1	ethanol	static freezing	2.5	2.5
S2	water	static freezing	2.5	2.5
S3	ethanol	stirred freezing	2.5	2.5
S4	water	stirred freezing	2.5	2.5

### X-ray Scattering

**Figure 2:**
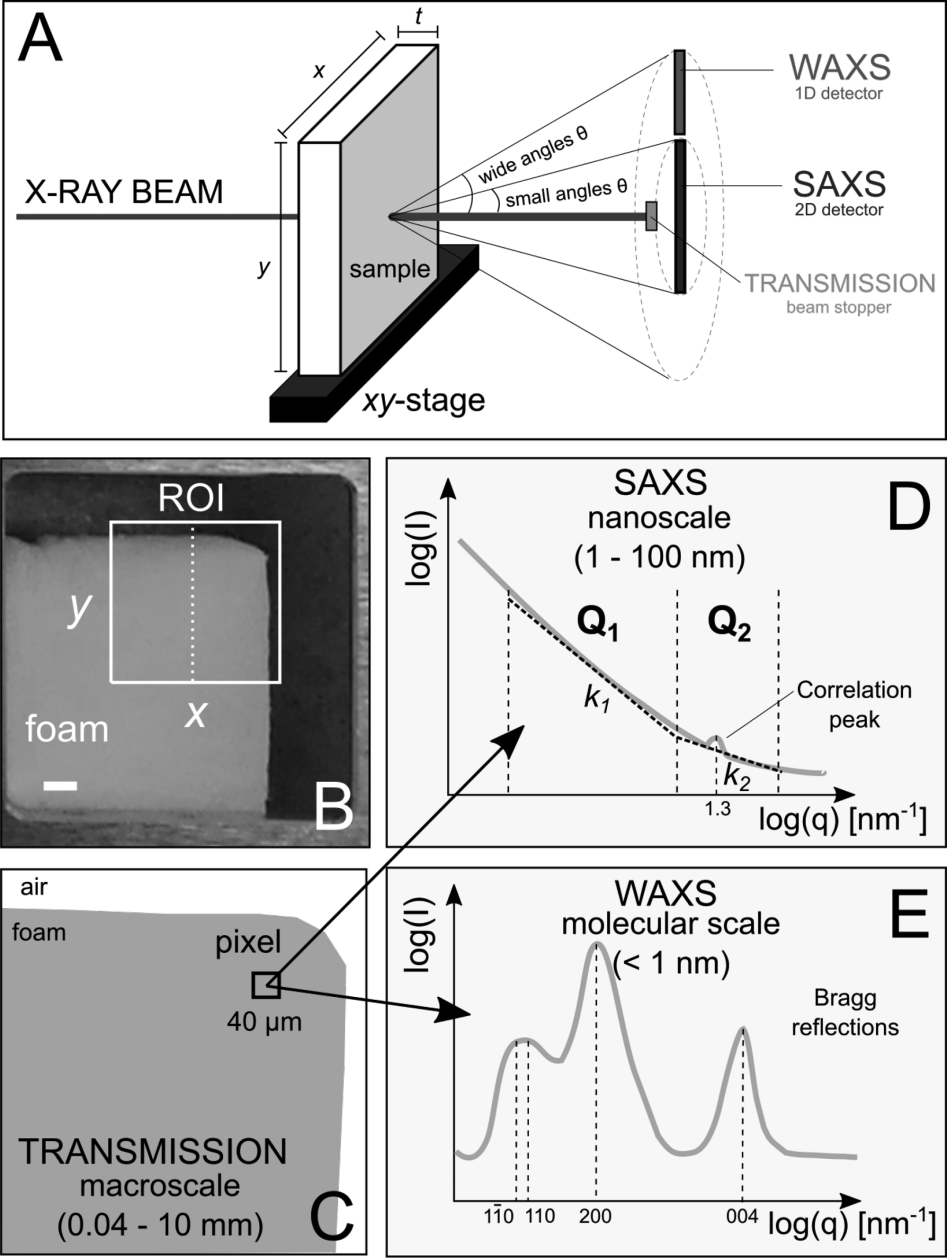
(A) Basic schematic of a scanning X-ray scattering experiment. A foam slice with thickness (*t*) of 3 mm is scanned by an *xy*-stage in front of the beam, along *x* and *y*. For each scanning point, a scalar value is recorded for the transmission at the beam stopper photodiode, as well as a 2D SAXS and a 1D WAXS pattern of the scattered intensity (*I*) as a function of the scattering angle (θ). Transmission, SAXS, and WAXS maps are generated. (B) Camera picture of the foam slice, indicating the region of interest (ROI) of 10 × 10 mm^2^, which is measured by sSWAXS with a beam of 40 × 40 μm^2^ (pixel size). The final map dimensions are [*x*, *y*] = [250, 250] pixels. Scale bar: 2 mm. (C) Schematic of the transmission maps with a resolution of 40 μm, which covers the macroscale. (D) Radially integrated SAXS curve showing the scattered intensity (*I*) expressed as a function of scattering vector (*q*). SAXS covers the nanoscale of 1–100 nm. On the basis of the slope in the logarithmic scale, two *q*-ranges are selected (Figure S2): *Q*_1_ (*q* = 0.1–0.8 nm^–1^, i.e., *d* ≈ 8–60 nm) and *Q*_2_ (*q* = 0.8–2.8 nm^–1^, i.e., *d* ≈ 2–8 nm). A correlation peak at *q* = 1.3 nm^–1^ is identified for some SAXS patterns (Figures S3, S4, and S5). (E) Integrated WAXS pattern indicating the Bragg reflections of cellulose crystallites (Figure S6 and Table S1 in the Supporting Information). WAXS covers the molecular scales smaller than 1 nm, similarly to diffraction.

The sSWAXS experiments were performed at the coherent small-angle X-ray scattering (cSAXS) beamline at the Swiss Light Source. The essential experimental setup that can enable multimodal scanning scattering measurements is simplified in Figure 2A. Briefly, an X-ray beam shines through the sample thickness, *t*, and is scattered by the electrons in the atomic shells of the matter within that scattering volume. After this interaction, signals from transmission, SAXS, and WAXS are simultaneously recorded, and a stage translates the sample in a scanning manner covering the [*x*, *y*] dimensions of the region of interest (ROI, Figure 2A). In our experiments, the X-ray beam is focused to 43 × 20 μm^2^ and the photon energy is set to 12.4 keV, which corresponds to a wavelength of λ = 1 Å. A photodiode, attached to the beam stopper, measures the intensity of the beam transmitted through the sample and generates a transmission map. Two-dimensional SAXS patterns are collected by a Pilatus 2M detector (1475 × 1679 pixels, pixel size = 172 × 172 μm^2^), and the one-dimensional WAXS patterns are collected by a Pilatus 300k detector (1475 × 195 pixels, pixel size: 172 × 172 μm^2^). The exposure time is set to 0.035 s for each data point, and the X-ray flux during the measurements is ∼5 × 10^11^ photons per second.

For these sSWAXS measurements, CNF-based foams are sliced to a thickness *t* = 3 mm by a vibration cutter. The slices are mounted with the *xy*-plane perpendicular to the X-ray beam direction (Figure 2A). The scanned ROI stipulates the [*x*, *y*] dimensions of the map (Figure 2B). sSWAXS is an imaging technique; thus, the image resolution is defined by the smallest value among the step size and the beam size. The step size determines the image pixel size. The map consists of 250 × 250 pixels, with a pixel size of 40 μm, scanned continuously along *y*. The step size is defined based on the beam size of 43 × 20 μm^2^. This map results in an ROI of 10 × 10 mm^2^. For each foam slice, a transmission map (62 500 transmission measurements), a SAXS map (62 500 SAXS patterns), and a WAXS map (62 500 WAXS patterns) are generated (one for each pixel of the image, Figure 2C). For SAXS (Figure 2D) and WAXS (Figure 2E), the scattered intensities (*I*) are radially integrated as a function of the scattering vector (*q*), defined by *q* = (4π/λ) sin θ, where λ is the X-ray wavelength and θ is half of the scattering angle. The sample orientation is only obtained from SAXS data because a 2D detector is necessary for the determination of the scattering pattern anisotropy. The codes used for the analysis were developed by the Coherent X-ray Scattering group at the Paul Scherrer Institute in Villigen, Switzerland, and can be found on the cSAXS web page at https://www.psi.ch/sls/csaxs/software. These scattering intensities are corrected by the X-ray transmission measured for the same pixel. We assume that air scattering has its own contribution to *I*(*q*); thus, it is subtracted as a background. The completeness of this data set provides high statistics and spatial resolution, and it requires statistical model-free data-analysis approaches.^45^ In our work we focus on statistical model-free analysis, although model-dependent SAXS analysis is also possible.^46^

In SAXS measurements (Figure 2D and Figure S2), the scattering vector range of *q* = 0.06–5.92 nm^–1^ covers structure sizes of *d* ≈ 1–100 nm. These scattered intensities follow a power law *I*(*q*) ∝ *q*^–*k*^, where the exponent *k*, corresponding to the slope in logarithmic scale, varies in two main *q*-ranges at different length scales: *Q*_1_, for *q* = 0.1–0.8 nm^–1^, i.e., *d* ≈ 8–60 nm; and *Q*_2_, for *q* = 0.8–2.8 nm^–1^, i.e., *d* ≈ 2–8 nm. WAXS (Figure 2E and Figure S6) provides information at the molecular scale, within the range of *q* = 6.91–31.73 nm^–1^, which covers structure sizes of *d* = 0.20–0.91 nm, i.e., <1 nm. For the correlation peaks in SAXS (Figure 2D and Figure S4) and for the Bragg reflections in WAXS (Figure 2E and Figure S6), Gaussian peaks are fitted to determine their full width at half-maximum (FWHM), maximum peak position, and intensity. The exponent and peak search analysis are developed for MathWorks MATLAB v2016b.

## Results and Discussion

### Hierarchical Structure of CNF-Based Foams

**Figure 3:**
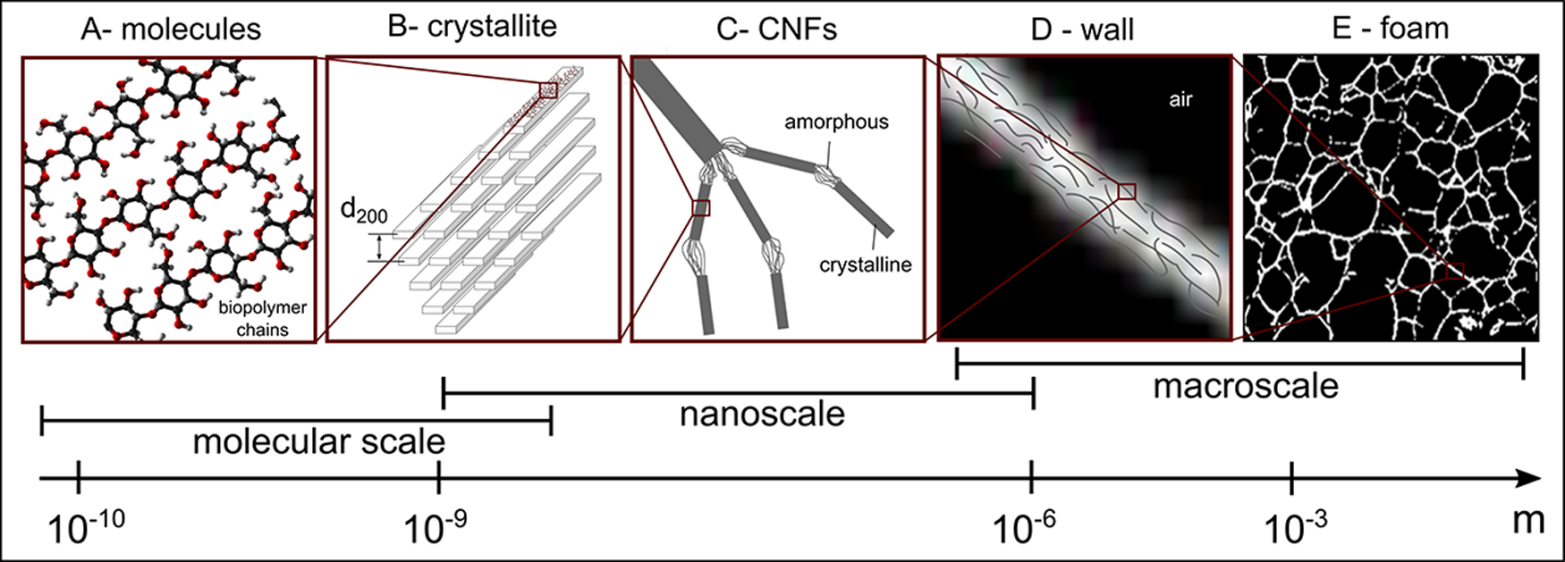
Definition of the multiscale hierarchical structure of CNF-based foams.

Figure 3 illustrates the multiscale hierarchical structure of CNF-based foams. At the molecular scale (Figure 3A), parallel cellulose biopolymer chains linked by hydrogen bonding^47^ form crystallites (Figure 3B). The diameter of such crystallites is in the nanometer range, separated by planes in the angstrom range.^48,49^ These crystalline structures alternate with amorphous ones and build cellulose nanofibrils (CNFs, Figure 3C). CNF diameters span over a wide range, as can be observed by the SEM images in Figure 1A, where the smaller fraction contains nanofibrils having diameters down to a few nanometers. The grinding process employed to create CNFs significantly increases the specific surface area, reaching values of 200 m^2^/g for supercritically dried CNFs. Such a high surface area confirms the high degree of fibrillation obtained with the grinding process. After the freeze–drying–thawing (FDT) process (Figure 1B and C), foams possess the characteristic ice-templated porosity, with larger pores in the range of 100–500 μm.^29^ Aggregated CNFs compose the walls of the foams (Figure 3D), which interconnect in a porous foam structure that spreads over the macroscale (Figure 3E). Due to fibril aggregation, a reduction of the specific surface area in the foams is expected, as confirmed by measurements (10–15 m^2^/g by krypton sorption and 4–10 m^2^/g by nitrogen sorption). A one order-of-magnitude reduction for the specific surface area of foams, compared to that of CNFs, is comparable with values reported by other studies in the literature.^27,50^ Over these multiple length scales, spanning from molecular to millimeter scale, the structure of CNF-based foams is investigated by sSWAXS. Transmission, sSAXS, and sWAXS maps are presented and discussed, covering the macroscale (0.04–10 mm) based on a microscopy approach, down to the molecular scale (<1 nm) based on WAXS. The presented results demonstrate commonalities and differences for four selected samples (S1–S4, Table 1), which have undergone different fabrication processes.

### Transmission Maps: Macroscale (0.04–10 mm)

**Figure 4:**
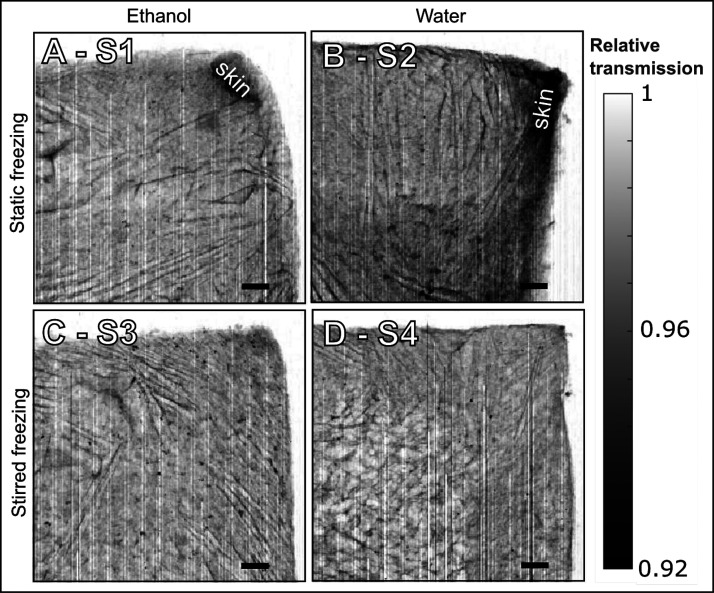
Relative transmission maps. The relative transmission is normalized by the air transmission. The vertical stripes are caused by variations in X-ray beam intensity. Scale bar: 1 mm.

Transmission maps provide overall insights on the foam structural organization based on the material composition and density, similar to that in diagnostics radiology. In Figure 4 we show the transmission maps, in which we observe differences in structural morphology on the millimeter length scale with a spatial resolution of 40 μm, determined by the step size in the scans. The vertical stripes in the transmission maps are caused by beam-intensity fluctuations. Few darker spots are also visible: the scattering signals from those areas do not show any change in structure, neither on the nanoscale, as evidenced in the SAXS curves, nor in the atomic structure, as observed in the WAXS curves (see Figure S1). Such darker spots may come from areas of higher local density in the sample. Although we do not expect contamination in these samples, we cannot exclude that these areas of lower transmission arise from contamination. A dense skin effect, visible mainly in samples S1 and S2 produced by static freezing (Figure 4A and B), is a consequence of higher material density, which is detected by the lower transmission of X-rays. The skin effect is significantly reduced in samples S3 and S4, which undergo stirred freezing. For these samples, the stirred freezing helps to redistribute the ice crystal more homogeneously; as a result, the cellulose structure is also more homogeneous and less sensitive to structural collapse at the surface, which is instead observed in the formation of a dense skin in sample S2. Furthermore, the surface tension, σ, of the solvent plays a role. Drying the foams from ethanol (σ = 22 mN/m) significantly decreases the wall capillary collapse, mainly due to lower surface tension compared to water (σ = 73 mN/m). At static conditions, the dense skin covers ∼2% of the ROI when thawed in ethanol (Figure 4A, single-sample evaluation). In water, this skin region covers ∼15% of the ROI (Figure 4B, single-sample evaluation), confirming the influence of drying the foam from a low-surface-tension solvent on the overall morphology.^29^ The influence of the freezing front direction on the structure is discussed later based on SAXS data.

### sSAXS Maps: Nanoscale (1–100 nm)

X-ray scattering at small angles provides structural information concerning long-range order at the nanoscale. Here, sSAXS is analyzed as spatially resolved maps of the CNF-based foams regarding (i) the structural compactness of CNF-based foams, which provides information on the fractal structure of the material based on the slope of the scattered intensity, *I*(*q*); (ii) the structural orientation of CNFs within the foam walls; and (iii) the molecular packing of CNFs, providing information on the CNF dimensions. As such, (ii) and (iii) provide specific information on the foam walls with a characteristic thickness in the range 1–10 μm, as observed using SEM imaging.^29^

#### Structural Compactness of CNF-Based Foams

Representative scattering curves *I*(*q*) of the CNF-based foams are shown in Figure S2. These multiple *I*(*q*) curves correspond to 50 *y*-positions along the centerline of the ROI (*x* = 125, Figure 2B). The logarithm of the scattered intensity, *I*, is plotted as a function of the logarithm of the magnitude of the scattering vector, *q*. Apart from particle size and shape, the slope, *k*, of scattering curves provides information on local particle heterogeneity, such as the surface roughness of CNF aggregates, based on the estimation of surface fractal dimensions.^51^ It has been reported for CNF^48,49,52,53^ that the *q*-range *q* < 0.9 nm^–1^ corresponds mainly to the scattering of the air–material interface, whereas *q* = 0.8–2.3 nm^–1^ corresponds to the scattering from the inner structure of the CNF aggregates. The range of *q* > 2.3 nm^–1^ mainly signals amorphous structures. We follow these known *q*-ranges to analyze the different phase contributions to the SAXS curves. The relation between *I* and *q* in SAXS obeys a power law with similar trends within two main *q*-ranges (Figure S2), defined as *Q*_1_ at the air–material interface (*q* = 0.1–0.8 nm^–1^, i.e., *d* ≈ 8–60 nm) and *Q*_2_ from the inner structure of CNF aggregates (*q* = 0.8–2.8 nm^–1^, i.e., *d* ≈ 2–8 nm).

**Figure 5:**
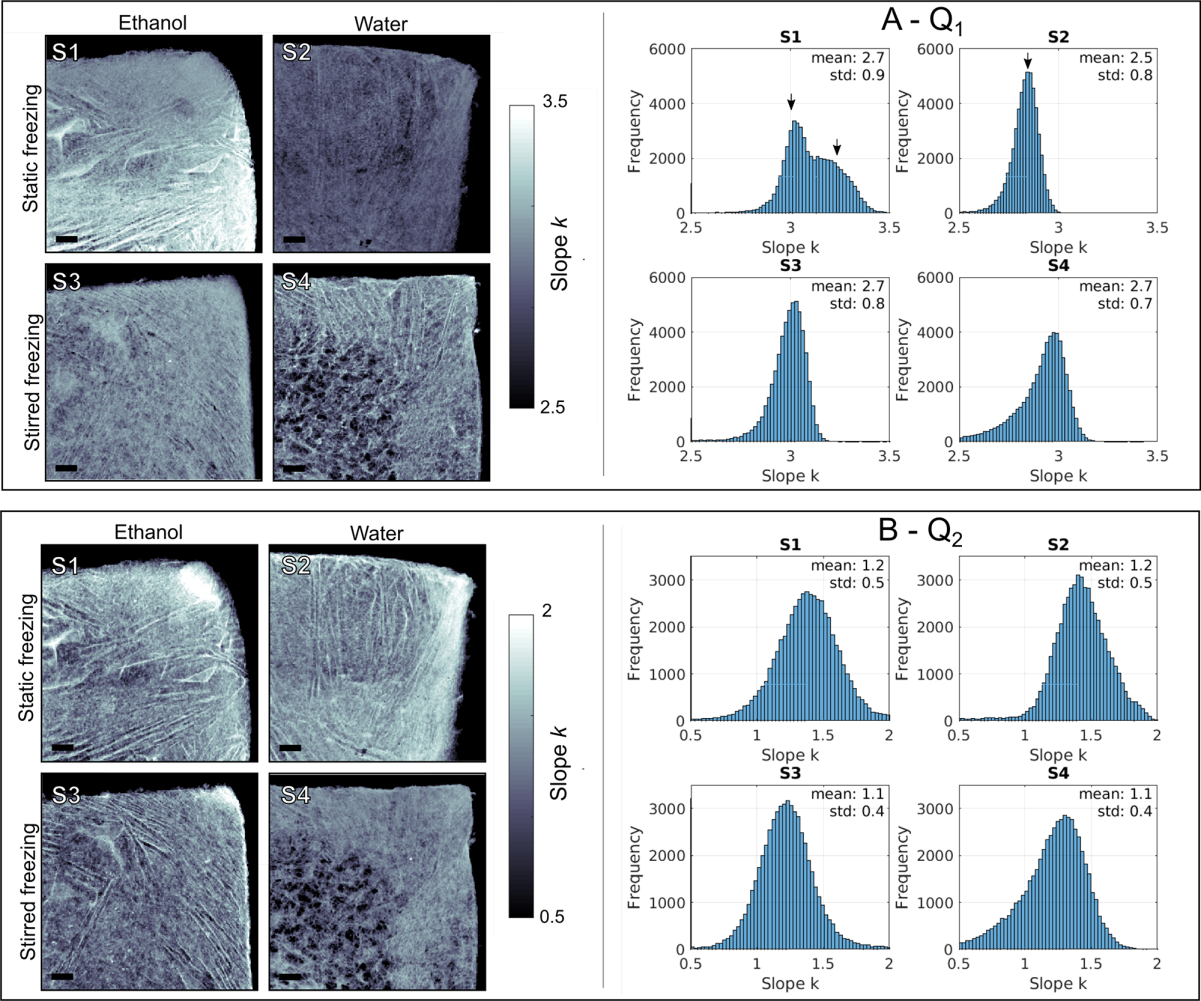
Slopes of SAXS signals divided in two main ranges. (A) *Q*_1_: maps of distribution of calculated slopes *k* (left) and histograms (right). (B) *Q*_2_: maps of the distribution of calculated slopes *k* (left) and histograms (right). Scale bar: 1 mm.

We calculate *k* for all scattering curves in these *q*-ranges, forming the maps of slope distribution shown in Figure 5. We assume that the slope in range *Q*_1_ is mainly related to air–material interface scattering, while the slope in *Q*_2_ contains internal structural information on the cellulose nanofibrils. Typically, *k* ≈ 1 denotes rodlike structures, and *k* ≈ 2 denotes a lamellar or planar structure for the form factor. When located in the range 2 < *k* < 3, the slope denotes mass fractals, while in the range 3 < *k* < 4, it indicates surface fractals with dense, homogeneous inner structures and surface roughness.^8^ Values of *k* ≈ 4 indicate a Porod law region, where smooth, well-defined interfaces are present. The Porod region is not observed in our measurements (Figure S2), indicating that the interface between the CNF aggregates and air are neither sharp nor smooth in this solid state.

At the *q*-range *Q*_1_ (Figure 5A), the slopes within 2.5 < *k* < 3.5 indicate variations between mass and surface fractals. In general, scattering from surface fractals occurs only at the surface of the material, whereas the scattering from the mass fractals occurs at both the inner structure of the material and its surface.^51^ Mass fractal objects have a complex network of pores that penetrates the object and generates more scattering. This porosity depends on the aggregation state of structural units, in this case, the CNF building blocks. As we consider that in *Q*_1_ the surface between air and CNF is the main source of the scattering signal, we can assume that the foam walls have relatively low porosity, behaving mostly as surface fractals. It is noteworthy that sample S1 in Figure 5A has the largest *k* distribution among the measured foams. It is clear that one area of the sample has a higher wall porosity, which generates mass fractal scattering (*k* > 3). We confirm that these two distinct areas are indeed due to structural differences in the foam wall at the nanoscale, as the sample overall density remains constant at the macroscale, as shown in Figure 4A. This result highlights the importance of multimodal analysis for the structural characterization of hierarchical materials. Despite the relatively wide distribution of *k* values, there are clear differences in the histograms of samples S1 (ethanol) and S2 (water) in Figure 5A, which are indicated by arrows. The general trend toward higher *k* for sample S1 indicates an increase in the porosity of the foam walls with the thawing in ethanol after static freezing conditions, while sample S2 thawed in water remains as a surface fractal with lower wall porosity. Thawing in ethanol loosens the surface fractal CNF aggregates by expanding the space between the fibrils and transforming some regions of sample S1 into mass fractals. This difference in wall porosity is less noticeable for samples fabricated under stirred freezing conditions (S3 and S4 in Figure 5A), which are homogeneous.

In the *q*-range *Q*_2_ (Figure 5B), the *k* < 2 are mainly attributed to the CNF form factor, *P*(*q*). Values of *k* = 2 are related to 2D platelike objects, while *k* = 1 describes rodlike objects. The tendency of *k* → 2 is more pronounced in the skin regions, which are clear in the transmission maps (Figure 4). A higher *k* value also indicates a higher 2D aggregation degree of CNFs in such regions. The most structurally uniform foam is sample S3, which tends to have rodlike structures, i.e., *k* → 1. This confirms the macroscopic observation of the foam structural stability for sample S3 in our previous study,^29^ as measured by microscopy and oil-absorption tests.

#### Structural Orientation of CNF-Based Foams

**Figure 6:**
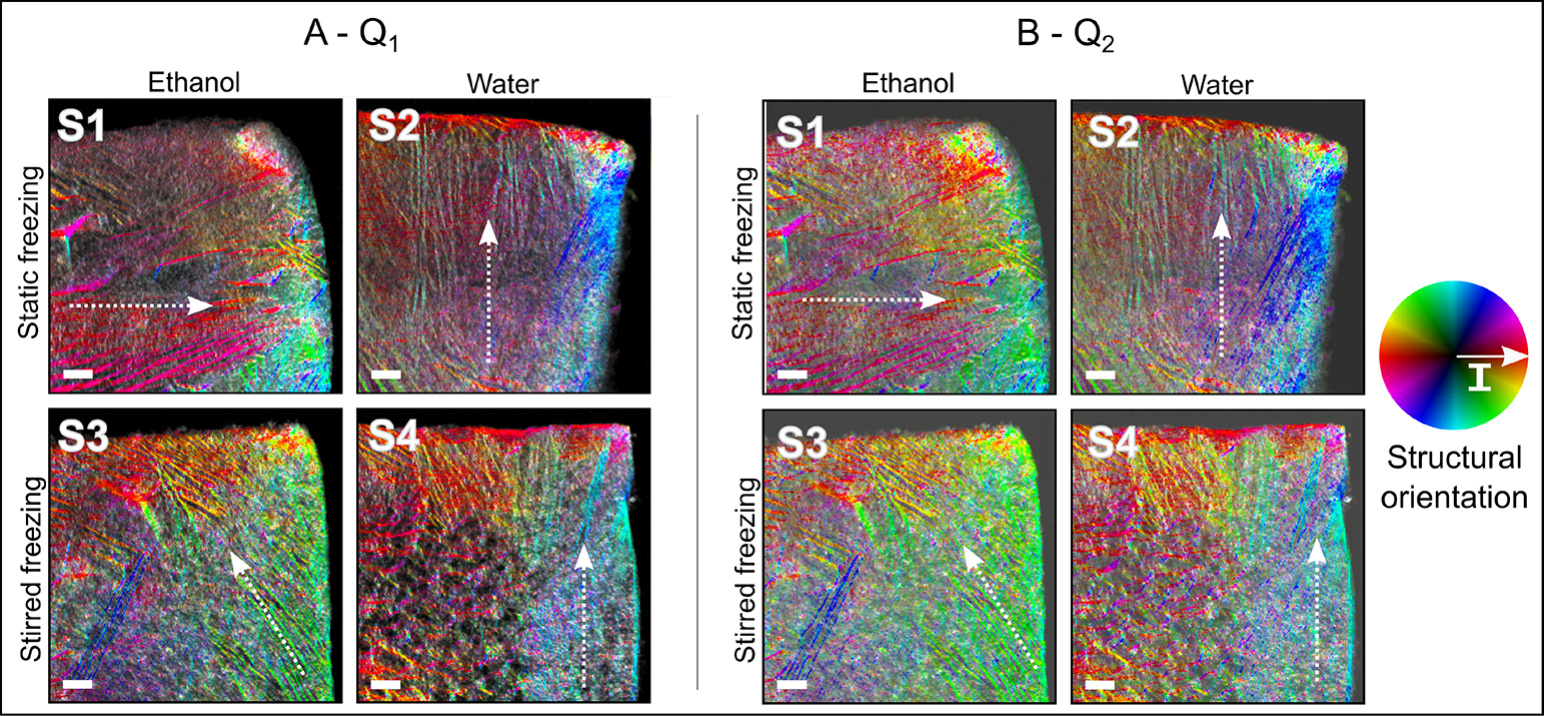
Structural orientation of CNF-based foams from sSAXS measurements. The color-wheel hue indicates the sample structural orientation, while the color-wheel brightness indicates its orientation intensity (*I*), i.e., the degree of orientation. (A) *q*-range *Q*_1_ including the orientation of structures with dimensions in the range of 8–60 nm. (B) *q*-range *Q*_2_ including the orientation of structures with dimensions in the range of 2–8 nm. The dotted arrows indicate the main direction of the freezing front. Scale bar: 1 mm.

We investigate the influence of freezing fronts imposed by the different FTD process steps on the structure of CNF-based foams by analyzing the anisotropy of the samples in the nano- and macroscale. For this orientation analysis of the foam structure, the fast online-analysis method developed by Bunk et al.^42^ is used. Briefly, the SAXS intensities, *I*(*q*), of two-dimensional scattering patterns are integrated into 16 azimuthal segments as a function of the azimuthal angle (ξ). The curve *I*(ξ) is approximated by a cosine function through a discrete Fourier transform. The average scattering intensity of the scattering pattern is represented by the baseline of the cosine, which is called the symmetric amplitude of the cosine function (*a*_sym_). In the case of anisotropic scattering, the intensity of a sample orientation is given by the amplitude of the cosine function, which is called the asymmetric amplitude (*a*_asym_). As a result, the degree of orientation can be calculated by the ratio *a*_asym_/*a*_sym_. The phase shift, ξ_s_, is directly related to the direction of the scattering-pattern orientation in reciprocal space, which is perpendicular to the structural orientation in real space. The result of such structural-orientation analysis is illustrated for *q*-range *Q*_1_, due to structures within 8–60 nm (Figure 6A), and for *q*-range *Q*_2_, due to structures within 2–8 nm (Figure 6B). The color-wheel hue indicates the orientation of the nanostructures in the sample in real space, while the color-wheel brightness indicates the orientation intensity (*I*), i.e., the degree of orientation averaged within each pixel.

The main differences in Figure 6 are observed between samples S1 and S2 (static freezing) and samples S3 and S4 (stirred freezing). In the case of static freezing, samples show a dominant freezing front direction (see arrows in Figure 6). This dominance is characteristic of static conditions because ice dendrites grow following a preferential direction. Note that sample S1 has a predominantly horizontal freezing front direction, while sample S2 has a vertical one. This difference is due to a 90° sample rotation, which is a consequence of the different sample mounting during the measurement. For both samples, the predominant freezing front direction is perpendicular to the surface exposed to air in the refrigerator (step IV of the freezing process) on which the heat transfer is maximum. Conversely, in the case of stirred freezing (S3 and S4), local structural orientations are observed, but there is a lack of overall dominating orientation. These samples exhibit a grainlike structure, which is characteristic of stirred freezing. Ice nuclei grow as the CNF suspension is continuously stirred (until ∼50% of the suspension is frozen, and then the freezing is completed statically in a mold), so that each ice crystal can have an independent growth direction. As a result, pores and walls in the final material have the same orientation within the region where a single ice crystal has grown, but no overall dominating orientation. This structural orientation is observed at the nanoscale based on the SAXS signal measured per pixel, while the overall view of the sample is enabled by the scanning and mapping of the sample at the macroscale. Such conclusions are only possible by nondestructive techniques that allow multiscale structural characterization, such as sSWAXS.

Furthermore, this orientation analysis highlights the boundary effects. At the sample surface, CNF aggregates are oriented parallel to the sample surface and perpendicular to the freezing front direction. This effect is clear especially in samples S2 and S4, thawed in water. The boundary effect is related to the formation of a dense skin due to capillary collapse of CNF aggregates, which complements the differences in sample density highlighted by the transmission maps in Figure 4.

#### Packing of CNF-Based Foams

The scattered intensity is mainly composed of contributions from the form factor (*P*(*q*) = size and shape of particles) and the structure factor (*S*(*q*) = interaction between particles). As the intensity *I*(*q*) is always the convolution of *P*(*q*) and *S*(*q*), the form factor can only be measured in monodisperse systems without interparticle interactions. In these cases, the structure factor is *S*(*q*) = 1, such as in very dilute suspensions of identical particles. When anisotropic particles are closely packed together, their packing resembles an ordered lattice with center-to-center spacing approaching their diameter: as a result, the structure factor dominates the scattering curve, resulting in a correlation peak.^54^ Note that a perfectly regular lattice is not required to form a correlation peak. Simply, if anisotropic particles, such as CNFs, are uniformly oriented in 1D, then both the form and structure factors will contribute to the scattering intensity. The scattering intensity of CNF solutions is dominated by the form factor. In solution, the contribution of *S*(*q*) is often not observed because there is no clear particle orientation nor stacking.^1^

Through the signal segmentation of the large data set created by sSWAXS,^45^ we identify 2D scattering patterns with symmetric anisotropic arcs (Figure S3). When radially integrated, these arcs generate sharp correlation peaks at *q* = 1.3 nm^–1^ (Figure S4) that are located in specific regions of the sample. On the basis of the correlation peak position *q* = 1.3 nm^–1^, we could estimate a mean center-to-center distance (*d*) of 4.8 nm from *d* = 2π/*q*. This peak position is similar to other studies of cellulose where peaks were observed at *q* = 1.74 nm^–1^ by Kennedy et al.^54^ and at *q* = 1.6 nm^–1^ by Fernandes et al.^1^ These works measured CNFs in wet and solution conditions with relatively large beams, which cause the broadening of the correlation peaks. In our study, a sharper correlation peak could result from the dry ambient conditions of the sSWAXS measurements and from the small sample area per pixel (40 × 40 μm^2^). On the basis of these observations, we may assign this dimension *d* = 4.8 nm to the average distance between centers of neighboring CNFs, which would also indicate the maximum possible diameter for CNFs. In points where a correlation peak is observed, we assume that CNFs are well-oriented in 1D with nonuniform spacing.^55^Figure S5 highlights the [*x*, *y*] pixels where a correlation peak is observed in the SAXS signal.

When CNFs aggregate with some regularity in orientation, these correlation peaks could appear.^55^ If the CNFs are in close contact, there will be insufficient matrix material between the CNFs to provide enough SAXS contrast for a correlation peak to appear. This contrast depends on the type of solvent, as the proportion of crystalline cellulose and the arrangement of cellulose molecules in the CNFs influence the accessibility to water or other molecules.^39^ The occurrence of correlation peaks is more statistically relevant for samples S1 and S3, which are thawed in ethanol (Figure S5). We relate this observation to two main effects: (i) the weaker capillary forces exerted by ethanol, as compared to water; and (ii) the stronger interaction of water with cellulose, which promotes partial diffusion of water inside the cellulose nanofibril bundles.^8^ As a result, the correlation peak remains after thawing in ethanol and drying. These results complement the higher foam wall porosity described by the slopes and mass fractals for the samples thawed in ethanol (Figure 5) and confirm that the type of solvent used for thawing influences the orientation and packing of CNFs.

To our knowledge this is the first report of sharp small-angle scattering correlation peaks in systems containing only CNFs. The dimension *d* = 4.8 nm appears consistent with the upper limit for CNF diameter, and we suggest that this correlation peak may come from a 1D stacking of CNFs. Even though the form factor also contributes to *I*(*q*), the analysis of such correlation peaks could be used as a method to estimate the upper limit to the physical diameter of CNFs. This methodology would be more direct than either the Scherrer dimensions from WAXS or the surface-to-volume ratio from spectroscopic measurements.

### sWAXS Maps: Molecular Scale (<1 nm)

Information at the molecular scale enables verification of the environmental conditions, additives, or cellulose treatment that can alter the structure of the crystalline components of CNFs. The peak positions of our measurements are consistent with previously reported values for cellulose I (Table S1).^56^ Minor differences in peak positions among studies result from more disordered or distorted forms of cellulose crystallites.^54^ For example, the position of the (200) reflection shifts to higher scattering vectors *q* with increasing moisture content in the sample.^57−59^ This shift occurs independently of the cellulose crystallite orientation, suggesting that quantitative comparative analysis of peak positions needs to be performed under the same moisture levels.

In our data set, the WAXS signals of all data points are similar among all samples (Figure 2E, Figure S6, and Table S1), indicating that no structural changes occur at the molecular scale and that assembling and processing of CNFs into a foam does not influence its crystalline phase. Three peaks are observed. The first broad peak is related to the reflections of the cellulose crystallites (11̅0) at 10.7 nm^–1^ and (110) at 11.9 nm^–1^. They appear to be merged, similarly to the peaks of cellulose I.^54^ The second peak at *q* ≈ 15.9 nm^–1^ is related to the (200) reflection and shows itself as a well-resolved reflection. The third peak at *q* ≈ 24.4 nm^–1^ is related to the (004) reflection and has a high degree of broadening. Note that histograms of the peak positions in Figure S7 have very similar distributions and low standard deviations, confirming that the different foam processing does not influence the crystalline phase of CNFs. Also, the WAXS signal, showing no characteristic peak of crystalline urea, confirms that urea is completely washed away during the thawing step.

#### Dimensions of Cellulose Crystallites

The dimensions of cellulose crystallites are usually determined from WAXS reflections. The crystallite length is calculated along the cellulose chain by using the meridional reflection (004), while the crystallite width is calculated perpendicular to the chain direction by the equatorial reflection (200) (Figures 2E and 3B).^47^ We analyze only reflection (200), because the reflections (11̅0), (110), and (004) are either too broad or not well-resolved.

The Scherrer equation is applied to extract the cellulose crystallite width *W*,
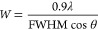
1where λ is the wavelength of X-rays and FWHM is the full width at half-maximum of reflection (200).^54,56^ The FWHM is obtained from the fitting of a Gaussian curve to reflection (200) (Figure S7). Uncertainties in the determination of *W* rise from other contributions to peak broadening apart from the size of the crystallites and instrumental defects, such as internal lattice defects and possible strains in the sample.^47,54^

The fitting of 250 000 reflections of the (200) plane results in an average cellulose crystallite width of 2.84 ± 0.07 nm (Figure S8). This width is inversely related to the number of diffracting lattice planes in the crystallite.^1^ Usually, (200) planes are separated by 0.4 nm, which is the distance of hydrogen-bonded sheets of cellulose in a crystallite.^56^ On the basis of the fitted average position of the (200) peak *q* ≈ 15.8 nm^–1^ (Figure S7A), a distance *d*_200_ of ∼0.40 nm is confirmed perpendicularly to the cellulose chain direction. This dimension is indicated in Figure 3B as *d*_200_. This crystallite width of 2.84 ± 0.07 nm corresponds to crystallites formed by ∼8 planes separated by *d*_200_ along the [200] direction.^1^ Even though the crystalline length is not well-resolved, the weak appearance and/or absence of the (004) reflection suggest that CNFs have longitudinal disorder in the foam structure.^56^ The average position of the (004) peak *q* ≈ 24.4 nm^–1^ (Figure S7B) corresponds to a distance *d*_004_ of ∼0.26 nm, which is the distance between the (004) planes.

## Conclusions

In this study, we investigate the hierarchical structure of CNF-based foams produced by the freeze–thawing–drying process, which has the potential for enabling scalability of cellulose-based materials. The sSWAXS method is applied for such multiscale structural characterization, and multimodal data correlate the macroscale morphology of the foams to the nano- and molecular-scale features of aggregated CNFs.

At the macroscale, spatially resolved transmission maps provide information on the foam morphology and density distribution over a millimetric area with a resolution of 40 μm. For static freezing conditions, a dense skin is formed at the surface of the sample. This skin effect is more noticeable for samples thawed in water versus those in ethanol, as the lower surface tension decreases the capillary collapse while drying. For stirred freezing conditions, the morphology and density of foams are more homogeneous, and fewer differences are seen among the solvent types.

At the nanoscale, the small-angle X-ray scattering (SAXS) analysis provides information on the structural compactness, structural orientation, and packing of CNFs in the foam walls. The structural compactness is analyzed based on the scattering curve slopes *k* and their related fractals. The type of solvent influences wall packing under static conditions, and samples thawed in ethanol have distinct macroscopic regions related to surface fractals and mass fractals. We confirm that the most structurally uniform foam is sample S3, which undergoes stirred freezing and is thawed in ethanol. On the basis of SAXS pattern anisotropy, the orientation of CNFs in the foam walls enables the correlation to the freeze front direction. Here, we emphasize the importance of a multimodal and multiscale approach for structural characterization: the presence, or lack, of a dominating foam orientation can only be determined if large areas of the sample are measured with high resolution. We measured a correlation peak that may represent the packing of CNFs in the foam walls. The *q* position of this peak enables the calculation of a center-to-center distance of 4.8 nm, which could be the upper value for the CNF diameter. Under both static and stirred freezing conditions, samples thawed in ethanol present more correlation peaks. We assume that ethanol can penetrate more between CNFs, enabling their reorganization and, at the same time, enhancing the SAXS contrast.

At the molecular scale, the Bragg reflections of cellulose are fitted by Gaussian curves in the wide-angle X-ray scattering (WAXS) signals. All samples show similar reflections, indicating that no changes occur at the molecular arrangement of cellulose due to the different processing routes. We measure a cellulose crystallite width of 2.84 nm composed by 8 planes separated by ∼0.4 nm. Along the cellulose chain direction, we measure a plane distance of 0.26 nm. These dimensions and reflections identify our CNFs as cellulose I type.

We show that sSWAXS allows the simultaneous investigation of the material macro-, nano-, and molecular-scale structures, providing spatially resolved information on the material distribution. We demonstrate that multimodal and multiscale structural characterization of hierarchical materials, such as cellulose nanofibrils and other self-assembled materials, e.g., polysaccharide complexes,^60^ is essential to understand and optimize the processing impact on the macroscopic properties.

As an outlook, future work would need to include larger statistics with more samples and combine the study with other characterization techniques, e.g., tomography or light scattering, in addition to optical and SEM imaging.

## Supporting Information Available

Typical values for WAXS reflections of cellulose, representative SAXS curves, SAXS curves showing the correlation peak, spatial distribution of the correlation peaks, representative WAXS scattering patterns, histograms of WAXS reflection Gaussian peak fitting, and calculated cellulose crystallite widths (PDF)
