# Supplementary material for: Hierarchical Structure of Cellulose Nanofibril-Based Foams Explored by Multimodal X-ray Scattering
Source: Biomacromolecules. 2022 Feb 23;23(3):676–86. doi: 10.1021/acs.biomac.1c00521 (PMC8924866; doi:10.1021/acs.biomac.1c00521)
Supplement: Supplementary file 1 — bm1c00521_si_001.pdf [file bm1c00521_si_001.pdf]

Supporting information: Hierarchical structure of cellulose nanofibril-based foams explored by multimodal X-ray scattering

Viviane Lutz-Bueno,[*,†,‡] Ana Diaz,[†] Tingting Wu,[¶] Gustav Nyström,[¶,‡] Thomas Geiger,[¶] and Carlo Antonini[*,¶,§]

†*Paul Scherrer Institute, Villigen PSI, Switzerland*

‡*Department of Health Sciences and Technology, ETH Zürich, Zürich, Switzerland*

¶*Laboratory for Cellulose and Wood Materials, EMPA Swiss Federal Laboratories for Materials Science and Technology, Dübendorf, Switzerland*

§*Department of Materials Science, University of Milano-Bicocca, Milano, Italy*

E-mail: viviane.lutz-bueno@psi.ch; carlo.antonini@unimib.it

Table S1: Scattering vector values, q, corresponding to cellulose crystal planes: comparison between values calculated using unit cell parameters from Ref.[3] and measurements in the present study. Note that values corresponding to $1\bar{1}0$ and 110 are approximate values, as the corresponding peaks are merged (see Figure S6).

Crystal plane	Ref.[3] q (nm^{-1})	Measurements q (nm^{-1})
$1\bar{1}0$	10.55	$\approx$ 10.7
110	11.82	$\approx$ 11.9
200	16.25	15.9
004	24.21	24.4

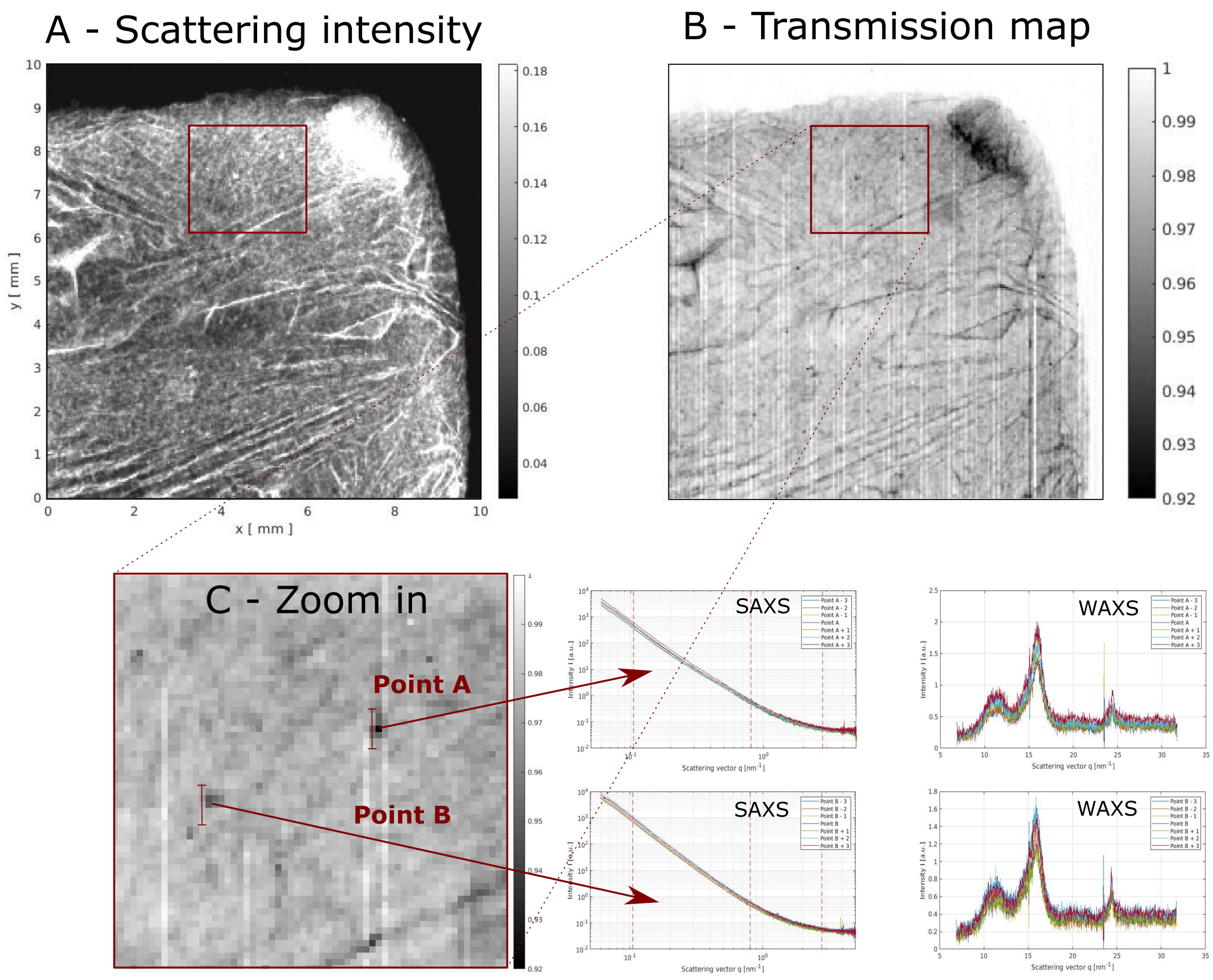

Figure S1: Sample S1. (A) Map of the symmetric scattering intensity. (B) Transmission map. (C) Zoom in the region with black spots. SAXS and WAXS curves around the Points A and B. Seven pixels were selected vertically along Point A and B. Note that there is no clear difference in the SAXS and WAXS curves, thus we associate these black dots to denser areas of the foam, even though the presence of impurities cannot be discarded.

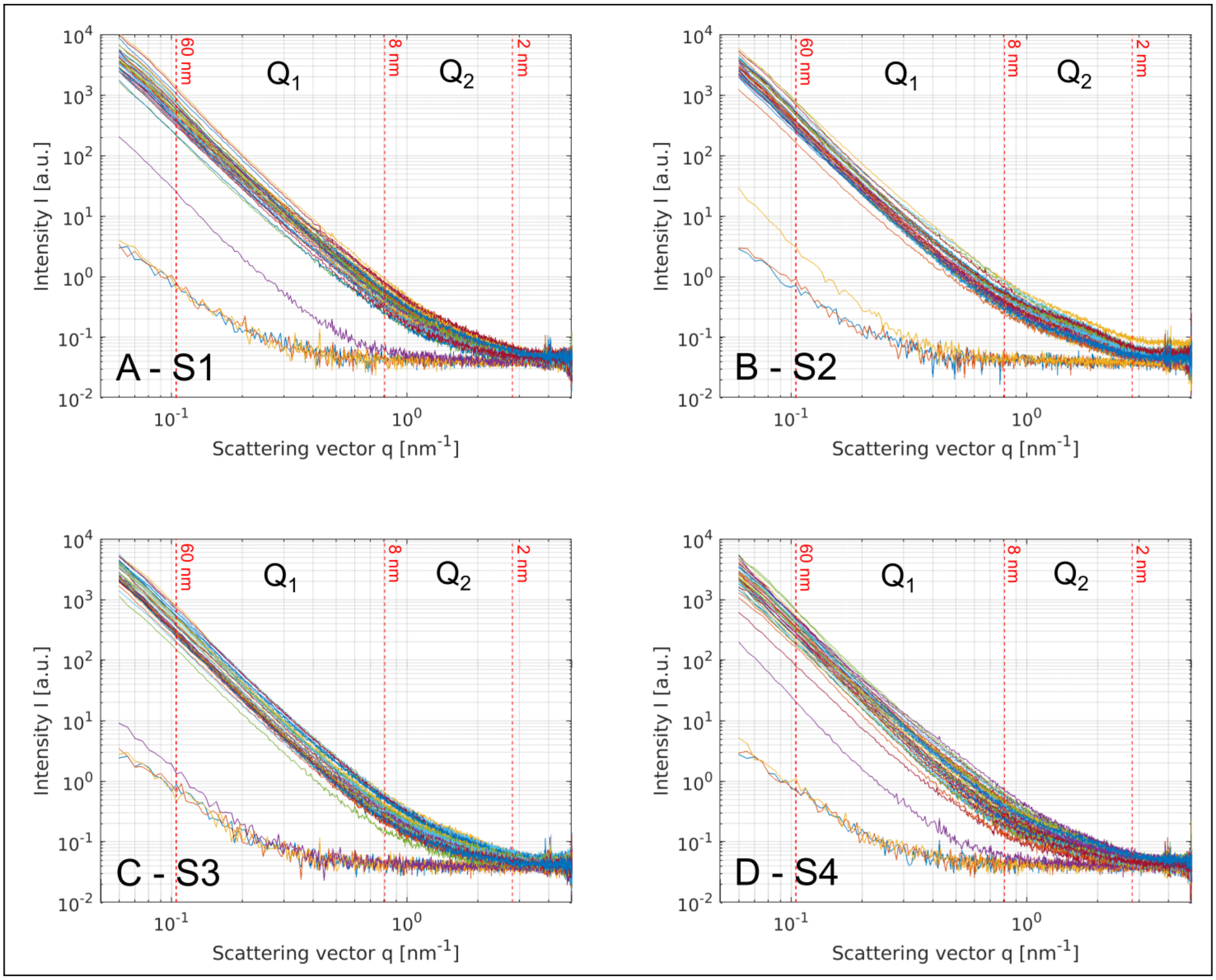

Figure S2: SAXS curves $I(q)$, intensity I as a function of the scattering vector q, for samples (A) S1, (B) S2, (C) S3, and (D) S4. Each plot contains 50 curves, located along the centerline of the sample (Figure 2B). Based on the slope k in logarithmic scale, two q-ranges are selected: Q_1 (q=0.1-0.8 nm^{-1}, i.e. $d \approx$8-60 nm), and Q_2 (q=0.8-2.8 nm^{-1}, i.e. $d \approx$2-8 nm).

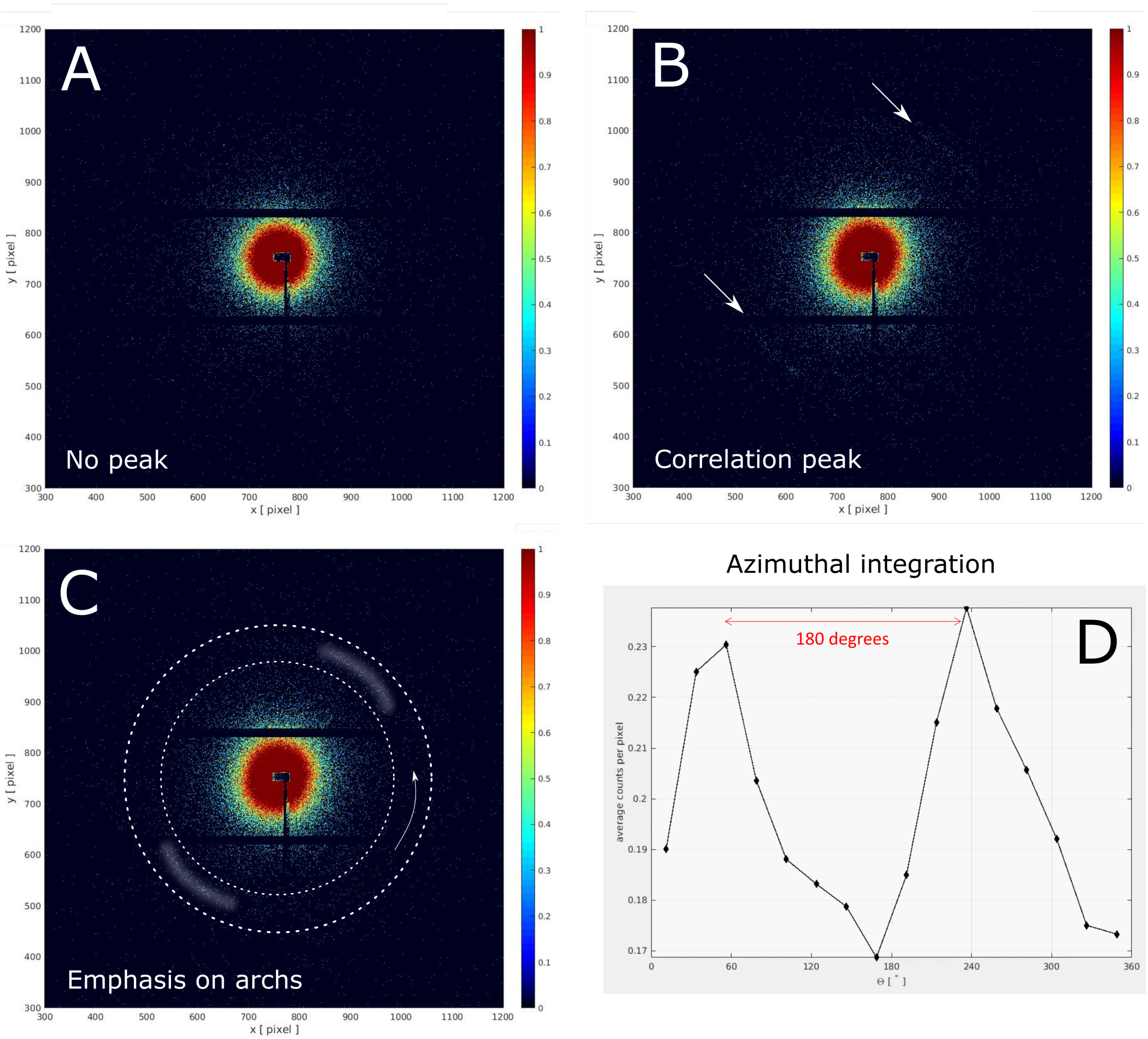

Figure S3: 2D scattering patterns showing the occurrence of the correlation peak. (A) 2D scattering pattern from a pixel without a correlation peak. (B) 2D scattering pattern with the formation of anisotropic arcs that are integrated into the correlation peak at $q = 1.3\ \mathrm{nm}^{-1}$. The arrows indicate the position of the arcs. (C) Due to the low counts on the detector, we put emphasis on the arcs from (B) and indicate the q-region selected for the azimuthal integration. (D) Scattering intensity as a function of the azimuthal angle θ, indicating the symmetry and anisotropy of the signal.

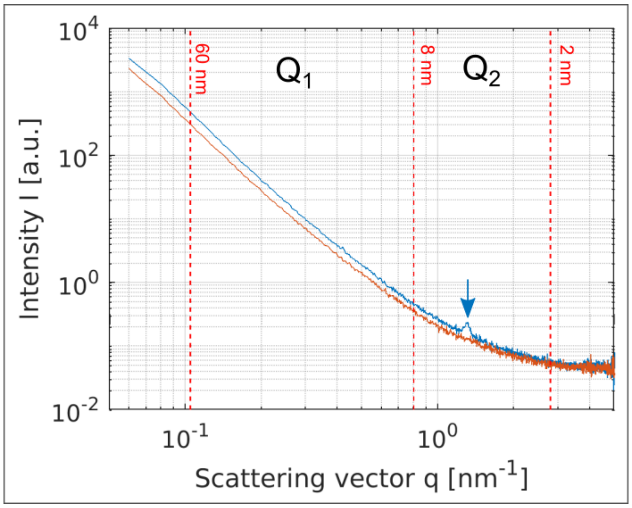

Figure S4: $I(q)$ curves with and without the correlation peak at $q = 1.3$ nm^{-1}. The peak position is indicated by the arrow. The presence and position of the correlation peak was determined for all $I(q)$ curves (250×250 curves per sample).

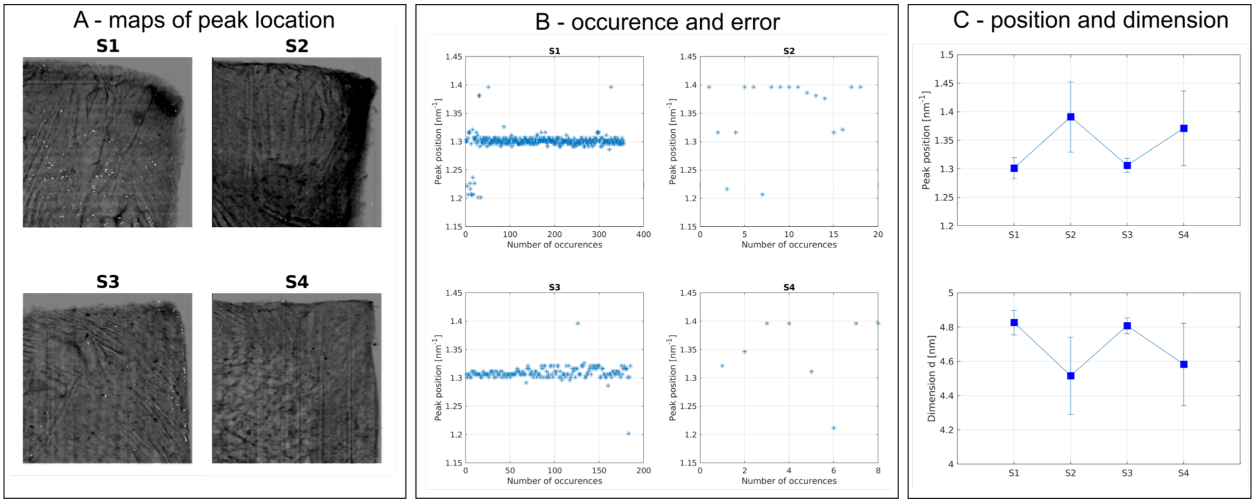

Figure S5: SAXS correlation peaks. (A) Spatial distribution of the correlation peaks' location, resulting from image segmentation.[1] The pixels that correspond to a $I(q)$ curve with a correlation peak are highlighted in white. These peak positions are overlaid with the transmission maps of the same samples. (B) Correlation peak position in q as a function of the number of occurrences. The samples thawed in ethanol (S1 and S3) exhibit clear prevalence of these correlation peaks peaks (hundreds of occurrences), while the presence of peaks can be neglected for samples thawed in water (S2 and S4), due to their statistical irrelevance (number of occurrences versus the total number of data points). (C) Top: average position of the correlation peak and the standard deviation. Bottom: structural dimension d that corresponds to the correlation peak position ($d = 2\pi/q$).

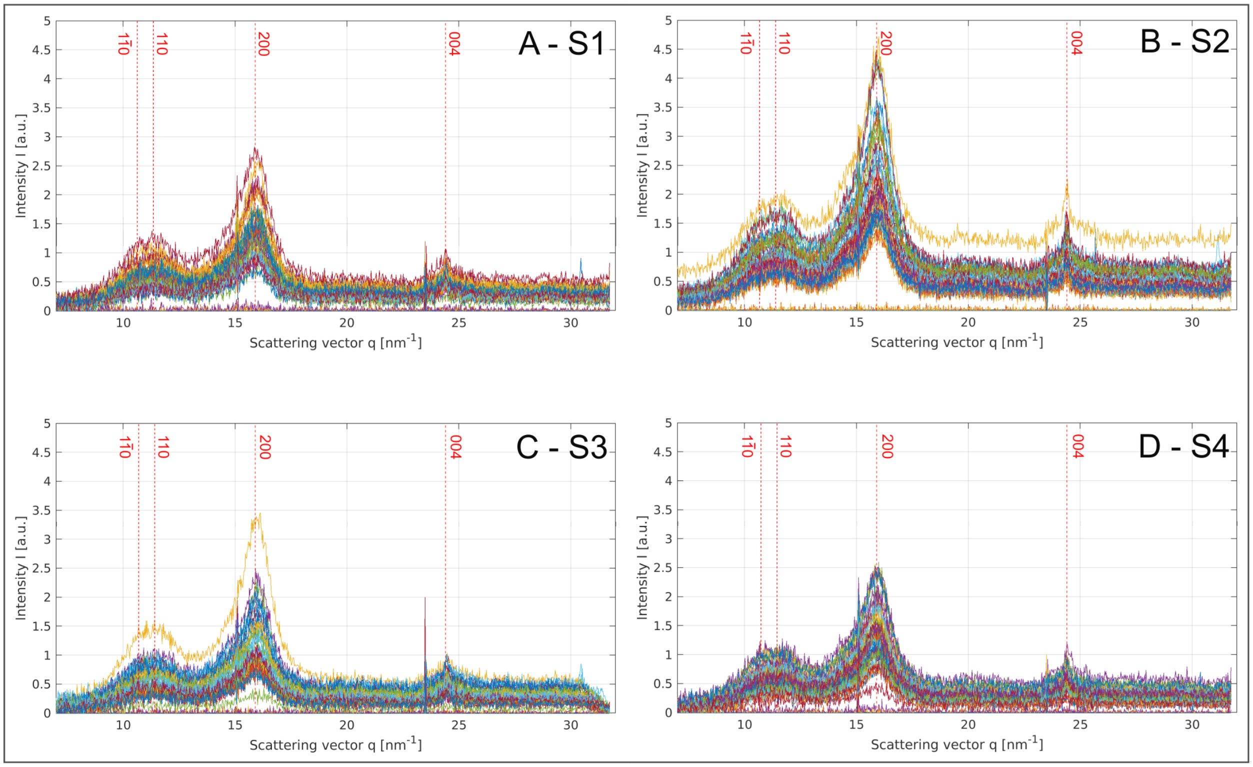

Figure S6: WAXS curves $I(q)$, intensity I as a function of the scattering vector q, for samples (A) S1, (B) S2, (C) S3, and (D) S4. Each plot contains 50 curves, located along the centerline of the sample (Figure 2B). Two peaks are identified: (200) and (004). The dimensions of cellulose crystallites are determined along the cellulose chain (length) by using the meridional reflection (004) and perpendicular to the chain direction (width) by equatorial reflection (200).[2]

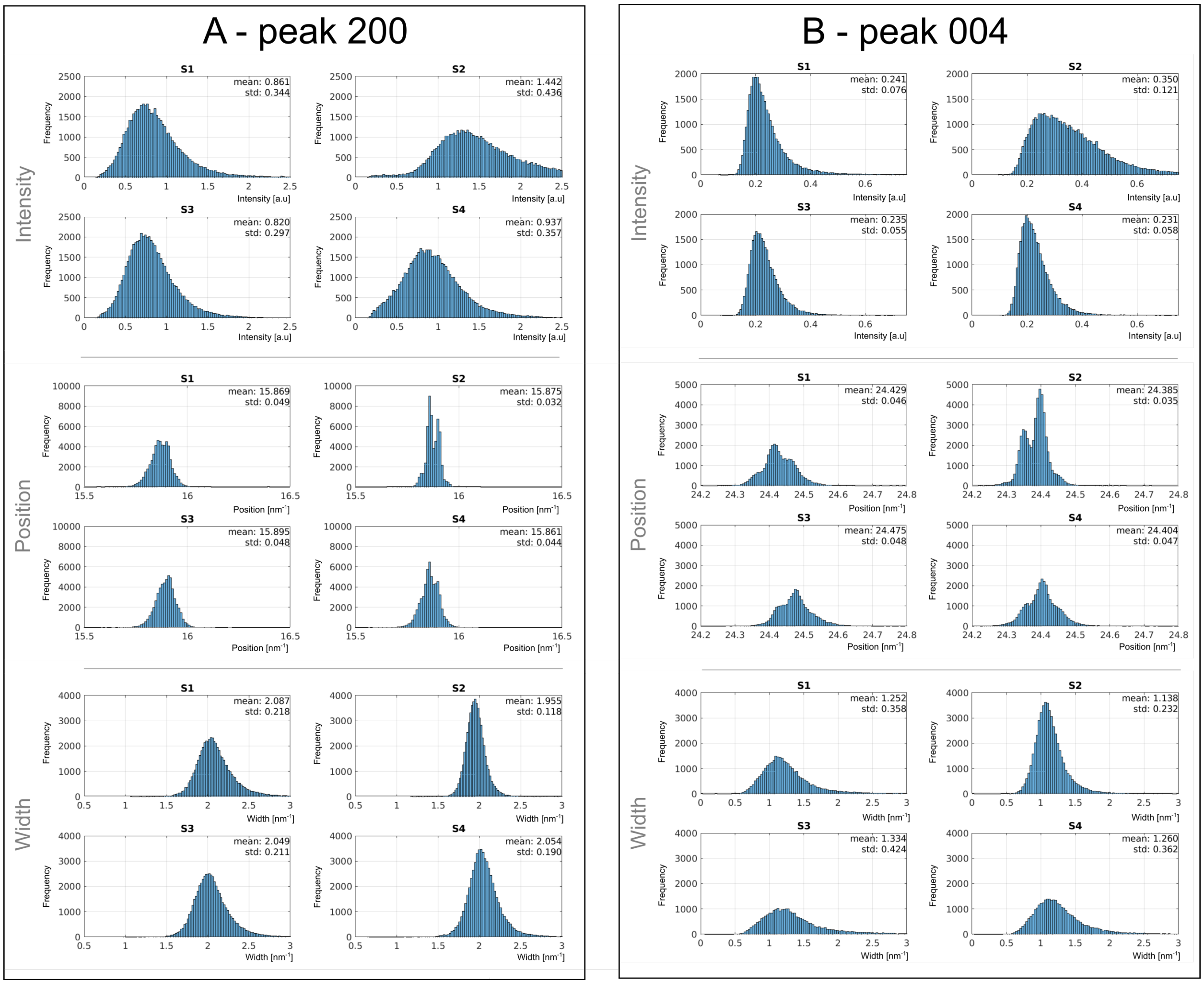

Figure S7: Histogram results of Gaussian peak fittings for the WAXS reflection on $I(q)$ curves. (A) Intensity (I), position (q), and width (FWHM) for 200 peak. (B) Intensity (I), position (q), and width (FWHM) for 004 peak. All the WAXS scattering curves in samples S1, S2, S3, and S4 are included.

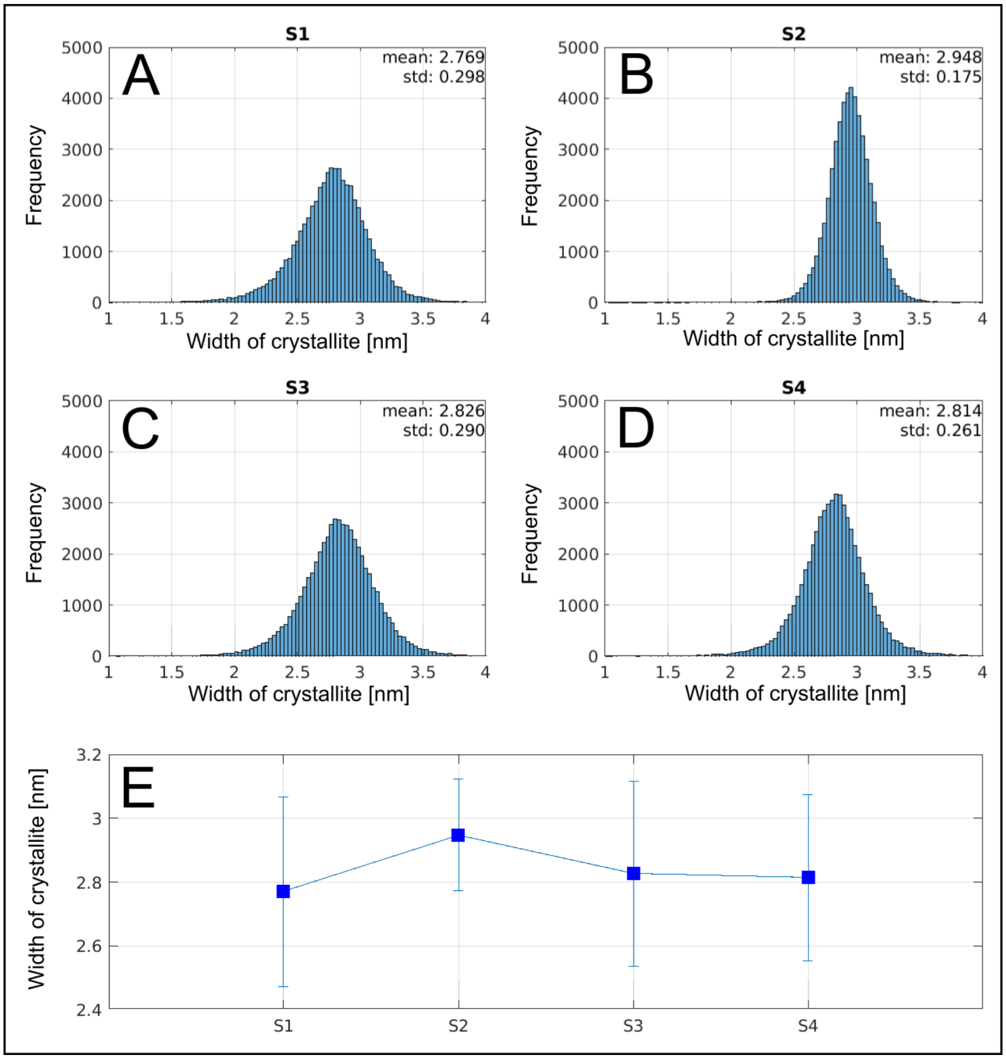

Figure S8: Width of cellulose crystallite calculated based on the Scherrer equation and 200 reflection. (A) S1, (B) S2, (C) S3, (D) S4, and (E) average width and standard deviations.

References

(1) Lutz-Bueno, V. et al. Model-free classification of X-ray scattering signals applied to image segmentation. *Journal of Applied Crystallography* **2018**, *51*, 1378–1386.

(2) Leppänen, K.; Andersson, S.; Torkkeli, M.; Knaapila, M.; Kotelnikova, N.; Serimaa, R. Structure of cellulose and microcrystalline cellulose from various wood species, cotton and flax studied by X-ray scattering. *Cellulose* **2009**, *16*, 999–1015.

(3) Nishiyama, Y.; Langan, P.; Chanzy, H. Crystal Structure and Hydrogen-Bonding System in Cellulose IÎ² from Synchrotron X-ray and Neutron Fiber Diffraction. *Journal of the American Chemical Society* **2002**, *124*, 9074–9082, PMID: 12149011.
